# The Role of Glutamine Homeostasis in Emotional and Cognitive Functions

**DOI:** 10.3390/ijms25021302

**Published:** 2024-01-21

**Authors:** Ji Hyeong Baek, Hyeongchan Park, Hyeju Kang, Rankyung Kim, Jae Soon Kang, Hyun Joon Kim

**Affiliations:** Department of Anatomy and Convergence Medical Sciences, College of Medicine, Institute of Medical Science, Tyrosine Peptide Multiuse Research Group, Anti-Aging Bio Cell Factory Regional Leading Research Center, Gyeongsang National University, 15 Jinju-daero 816 Beongil, Jinju 52727, Gyeongnam, Republic of Korea; baekbaek@gnu.ac.kr (J.H.B.); parkhc@gnu.ac.kr (H.P.); 2023210135@gnu.ac.kr (H.K.); rankyung@gnu.ac.kr (R.K.); jskang@gnu.ac.kr (J.S.K.)

**Keywords:** glutamine, glutamatergic neurotransmission, depressive disorder, cognitive impairment

## Abstract

Glutamine (Gln), a non-essential amino acid, is synthesized de novo by glutamine synthetase (GS) in various organs. In the brain, GS is exclusively expressed in astrocytes under normal physiological conditions, producing Gln that takes part in glutamatergic neurotransmission through the glutamate (Glu)–Gln cycle. Because the Glu–Gln cycle and glutamatergic neurotransmission play a pivotal role in normal brain activity, maintaining Gln homeostasis in the brain is crucial. Recent findings indicated that a neuronal Gln deficiency in the medial prefrontal cortex in rodents led to depressive behaviors and mild cognitive impairment along with lower glutamatergic neurotransmission. In addition, exogenous Gln supplementation has been tested for its ability to overcome neuronal Gln deficiency and reverse abnormal behaviors induced by chronic immobilization stress (CIS). Although evidence is accumulating as to how Gln supplementation contributes to normalizing glutamatergic neurotransmission and the Glu–Gln cycle, there are few reviews on this. In this review, we summarize recent evidence demonstrating that Gln supplementation ameliorates CIS-induced deleterious changes, including an imbalance of the Glu–Gln cycle, suggesting that Gln homeostasis is important for emotional and cognitive functions. This is the first review of detailed mechanistic studies on the effects of Gln supplementation on emotional and cognitive functions.

## 1. Introduction: Despite Being a Non-Essential Amino Acid, Glutamine Is Essential for Maintaining the Glutamate–Glutamine Cycle

The multifunctional amino acid glutamine (Gln) is a precursor of neurotransmitter amino acids—a major excitatory neurotransmitter, glutamate (Glu), and an inhibitory neurotransmitter, γ-aminobutyric acid (GABA)—and pivotal for normal brain energy metabolism and anti-inflammatory processes [1]. Gln is the most abundant non-essential amino acid in the central nervous system (CNS), where it is synthesized by astrocytes under normal conditions. However, Gln becomes conditionally essential in hyper-catabolic states and in tissues weakened by damage or stress [2]. These diverse characteristics have stimulated multiple studies on the roles and utilization of Gln under various conditions. This review focuses on the importance of Gln homeostasis in the pathology and treatment of emotional and cognitive disorders, particularly in terms of the maintenance of glutamatergic neurotransmission.

**Figure 1:**
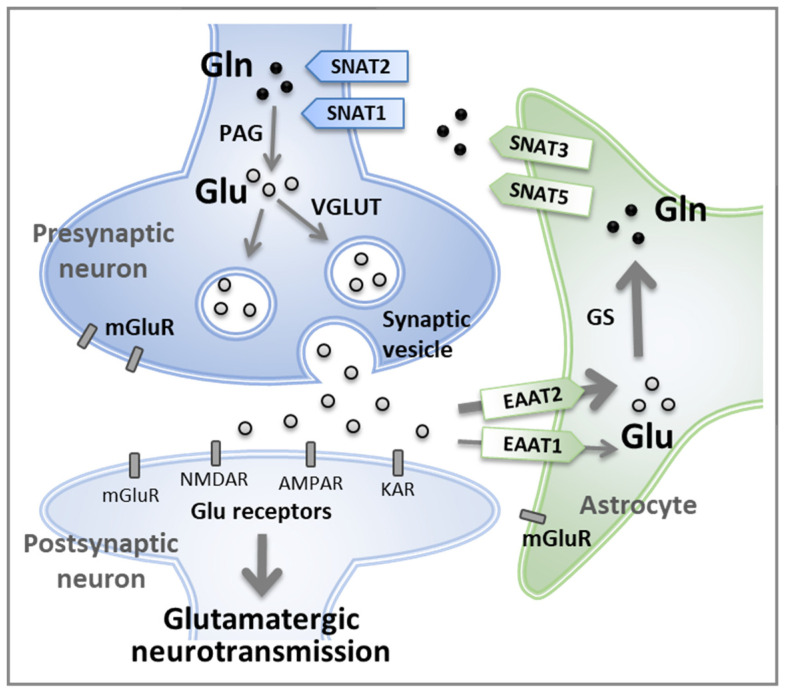
The glutamate–glutamine (Glu–Gln) cycle. High-affinity excitatory amino acid transporters (EAATs) in astrocytes, particularly EAAT2, clear Glu from the synaptic cleft. Astrocytes then convert Glu into Gln through glutamine synthetase (GS). Gln is released from astrocytes through sodium-coupled neutral amino acid transporter (SNAT)3 and SNAT5 and taken up by neurons through SNAT1 and SNAT2, where it is converted back to Glu by phosphate-activated glutaminase (PAG). This regenerated Glu is packaged into synaptic vesicles by vesicular glutamate transporters (VGLUTs) and released into synaptic cleft for subsequent excitatory neurotransmission. NMDAR, N-methyl-D-aspartate receptor; AMPAR, α-amino-3-hydroxyl-5-methyl-4-isoxazole-propionate receptor; KAR, kainite receptor; mGluR, metabotropic glutamate receptor.

Approximately 40% of all neurons are glutamatergic neurons, and 80–90% of all neurons have Glu receptors and are involved in excitatory functions [3,4]. Most glutamatergic neurons are distributed in the frontal cortex [4] and consume more than 80% of the total brain energy budget [5]. Gln participates in this glutamatergic neurotransmission, shuttling metabolic process and neurotransmitter pools via the Glu–Gln cycle, an astrocyte–neuron interaction pathway in the glutamatergic neurotransmission system [6]. Previous reviews have described the functional sequence of the Glu–Gln cycle in a glutamatergic tripartite synapse as follows [1,7,8,9] (Figure 1).

(1) Glu release: Neurons release the neurotransmitter Glu, which activates Glu receptors and generates an action potential. The released Glu can activate three different types of ionotropic receptors (N-methyl-D-aspartate receptors [NMDARs], kainate receptors [KARs], α-amino-3-hydroxyl-5-methyl-4-isoxazole-propionate receptors [AMPARs]) and metabotropic glutamate receptors (mGluRs), unlike other neurotransmitters that have one specific receptor [10]. The Glu receptors are located on membranes of presynaptic and postsynaptic neurons as well as on astrocytes. This receptor diversity contributes to Glu being the predominant neurotransmitter in the CNS.

(2) Glu uptake: After action potential is generated, Glu transporters rapidly uptake Glu from the synaptic cleft and turn off the signal to be ready for subsequent action potentials. In addition, this uptake prevents the accumulation of excess Glu in the synaptic cleft and protects neurons from excitotoxicity. Astrocytes express specialized proteins called Glu transporters, such as excitatory amino acid transporter (EAAT)1 (=GLAST) and EAAT2 (=GLT-1). Both EAAT1 and EAAT2 are densely distributed in astrocyte membranes facing neurons, and EAAT2 is the predominant Glu transporter. Some of the EAAT2 (approximately 10%) is found in the membrane of neurons [11].

(3) Gln synthesis: In astrocytes, Glu can be metabolized via the tricarboxylic acid cycle or converted into Gln by the enzyme glutamine synthetase (GS, EC 6.3.1.2). GS catalyzes the reaction that combines Glu and ammonia to form Gln. This conversion allows for the safe storage and transport of Glu in a non-toxic form.

(4) Gln release: Newly synthesized Gln is released from astrocytes via sodium-coupled neutral amino acid transporter (SNAT)3 and SNAT5, which are mainly expressed in astrocytes [12,13], and delivered to neurons via SNAT1 and SNAT2, which are mainly expressed in neurons [14,15,16].

(5) Glu regeneration: In neurons, Gln is hydrolyzed and converted back into Glu by the enzyme phosphate-activated glutaminase (PAG, EC 3.5.1.2). This regenerated Glu can be packaged into synaptic vesicles by vesicular Glu transporters (VGLUTs) after which it is readily available for the next excitatory neurotransmission.

Nuclear magnetic resonance (NMR) spectroscopy studies in rodents and humans using ^13^C-labeled glucose or acetate indicate that Gln mediates Glu recycling, and Glu provides 80–90% of the material for Gln synthesis in astrocytes [17,18,19]. Because Gln plays a pivotal role in the CNS, the imbalance of Gln homeostasis and the Glu–Gln cycle—e.g., through astrocyte ablation, Gln synthesis suppression, or inhibition of Gln transport into neurons—disrupts glutamatergic neurotransmission [1,20]. In addition, alterations in Glu–Gln homeostasis and glutamatergic neurotransmission are closely linked to a variety of pathological conditions in the brain and have garnered attention as a common cause of emotional and cognitive disorders [9,20,21,22].

Inspired by these findings, Gln supplementation experiments have been conducted to test whether depressive and cognitive disorders could be mitigated by exogenous Gln. Remarkably, as detailed in this review, Gln supplementation shows antidepressant effects and cognition improvements in both humans [23,24,25,26,27,28] and rodents [20,29,30,31]. In addition, Gln supplementation normalizes glutamatergic neuronal activity in rodent models of depressive disorder or mild cognitive impairment (MCI) with comorbid hypoactive glutamatergic neurotransmission [20,31,32].

These results highlight the importance of obtaining a detailed mechanistic understanding of the antidepressive and cognition-improving activities of Gln, which may lead to the development of novel therapeutics. The importance of the Glu–Gln cycle and glutamatergic neurotransmission in the pathogenesis of emotional and cognitive disorders has been reviewed elsewhere [1,7,8,9,33,34]. However, less attention has been devoted to the effects of Gln supplementation on glutamatergic neurotransmission and, in this regard, the beneficial effects of Gln on emotional and cognitive disorders. Therefore, in the following sections, we summarize the importance of Gln homeostasis on emotional and cognitive functions and the latest studies showing the protective effects of Gln supplementation on glutamatergic neurotransmission in the context of emotional and cognitive behaviors.

## 2. Glutamine Homeostasis Is Closely Related to Emotional and Cognitive Functions

The relationship between Gln homeostasis and emotional and cognitive functions is supported by clinical observations of altered Glu–Gln homeostasis and glutamatergic activity in patients with brain disorders accompanied by cognitive impairment, including major depressive disorder (MDD) [9,35,36,37,38], schizophrenia [8,39], bipolar disorder [40], and Alzheimer’s disease (AD) [34]. Reports that a variety of depression treatments, including antidepressants and electroconvulsive therapy (ECT), increase Glu or Gln levels in the medial prefrontal cortex (mPFC) of depressed patients [38,41,42,43,44] further strengthen the hypothesis that Glu–Gln homeostasis is closely related to emotional disorders.

Furthermore, postmortem studies in MDD and bipolar disorder patients have consistently demonstrated prominent decreases in astrocyte number and density within cortical regions [45,46]. Reductions in GS activity or expression levels have also been reported in the PFC of MDD and schizophrenia patients [47,48]. In the Glu–Gln cycle, astrocytes are responsible for the de novo synthesis of Gln via GS and for delivering Gln to neurons. Therefore, it could be assumed that a shortage of Gln caused by a decrease in astrocyte number and GS activity may be one of the causes of hypoactive glutamatergic neurotransmission [31]. This hypothesis is supported by data from rodent models showing that astrocyte ablation or the inhibition of GS or Gln transporters leads to reductions in both Glu–Gln levels and glutamatergic activity within the PFC, accompanied by increased depressive behaviors and cognitive impairments [20,22,31].

As noted above, various studies have examined Gln, Glu, or Glx (Glu + Gln) levels in emotional and cognitive disorder patients. However, the results are inconsistent across the studies. Even if the results are limited to studies of cortical areas in patients with depression, Gln or Glu levels, which are mainly analyzed using proton magnetic resonance spectroscopy (^1^H-MRS), are reported to increase [49,50], decrease [51,52,53,54], or remain unchanged [55,56]. These confusing results may be due to the vast heterogeneity in illness stage, medications, regions of interest, MRS acquisition parameters, and data processing methods [53,57]. In addition, creatine levels, which are usually used as an internal reference for MRS analysis, are lower in the brains of patients with depression [53], schizophrenia [58], and MCI [59] than in normal controls. Therefore, Gln and Glu MRS results for emotional and cognitive impairment patients may have been overestimated when creatine was used as an internal reference [53].

Considering these points, recent meta-analysis reviews estimate that the amount of Gln decreases in the brain or blood of patients with depression [53] or schizophrenia [39] but increases in the brain of patients with bipolar disorder [60]. These results are consistent with reports that GS expression levels decrease significantly in the cortical regions in postmortem brain samples of depression [61] and schizophrenia patients [48] but not in those of bipolar disorder patients [61]. Furthermore, decreased Gln contents in the PFC are consistently found in mouse models of depression and MCI induced by chronic stress [20,62] or phthalate exposure [63,64]. These animal model studies strengthen the results of the clinical and the postmortem studies that show that Gln levels decrease in the cortical region of patients with depression.

## 3. Regulation of Glutamine Concentration in the Central Nervous System

Gln is present at concentrations of 500–700 μM in plasma, occupying about 20% of the total free amino acids in plasma. Its concentration is 500–1000 μM in cerebrospinal fluid (CSF), 200–500 μM in extracellular fluid (ECF), and 5–7 mM in the whole brain [11,65,66,67]. Gln content in plasma, CSF, and ECF is similar, whereas the levels can vary for other amino acids. The even distribution of Gln facilitates Gln transport between the blood/CSF and ECF [1,68]. For substances with low concentrations to be transported into a high-concentration compartment, they must move against the concentration gradient. However, the Gln concentration gradient is relatively flat under normal physiological conditions, and Gln transporters are almost saturated due to a high concentration of Gln. Therefore, Gln can travel between blood and CNS compartments even without high-affinity transporters [68].

By contrast, Glu concentrations vary in different compartments of the body (50–100 μM in plasma; 9–14 mM in the whole brain), with low levels in the CSF (3–6 μM) and ECF (0.5–2 μM) [66,67,69]. Neurons, astrocytes, and the blood–brain barrier (BBB) help maintain these low Glu concentrations in ECF for normal neurotransmission. Specifically, Glu transporters (EAATs) operate in the BBB to remove Glu from the ECF, preventing net entry into the brain and maintaining low Glu levels [69].

The BBB, which is composed of endothelial cells of blood vessels, pericytes, and astrocyte endfeet, functions to isolate the bloodstream from the CNS to maintain the homeostasis of the neural environment [70]. The movement of nutrients, ions, and other molecules between the blood and brain is tightly regulated by the BBB. The BBB has two types of transporters: (1) efflux transporters, which transport a variety of lipophilic molecules from brain cells toward the blood [71], and (2) nutrient transporters, which highly selectively transport specific nutrients between the blood and brain cells [70]. Gln and Glu are transported via specific nutrient transporters to maintain specific intracellular and extracellular concentration gradients.

After Glu is released from presynaptic neurons, active synthesis of Glu occurs through the Glu–Gln cycle to maintain the high intraneuronal Glu concentration. Because the extracellular Glu concentration is much lower than that inside the neuron, Gln, which is abundant outside the cell, is taken up by neurons and utilized to synthesize Glu. The extracellular Gln originates from (1) Gln synthesized in astrocytes by GS and (2) Gln transported from blood vessels across the BBB to the brain [67].

Accumulating evidence shows that Gln concentrations in the brain and blood decrease under chronic stress, inflammation, and oxidative stress conditions [24,35,36,53,72]. In addition, under conditions of high energy demand, such as stress and inflammation, Gln in the brain is used for stress responses and energy metabolism [1,67]. Because the brain does not store Gln in it, Gln must be supplied from the blood to maintain Gln homeostasis in the CNS [73]. However, the Gln content in the blood is lower than that in the CNS and is not sufficient to meet the demand of the brain under conditions of high energy demand [74]. In these cases, it is necessary to supply exogenous Gln through diet or injection. Neutral amino acids, including Gln, can cross the BBB. However, acidic amino acids, such as Glu and aspartate, cannot cross the BBB as efficiently as Gln due to the small number of transporters in the plasma membrane [67]. Even if Glu is supplemented in the CNS, it must be converted to Gln to enter neurons. Moreover, Glu itself is a neurotransmitter and can be neurotoxic when oversupplied [1]. Therefore, supplying Gln rather than Glu is thought to be a safe way to preserve Glu–Gln homeostasis in the CNS.

## 4. Clinical Studies of the Effects of Glutamine on Emotional and Cognitive Functions

The effects of Gln supplementation on the human body have been primarily investigated in terms of recovery from acute Gln deficiency and muscle fatigue after exercise [75,76,77,78]. These studies focused on energy metabolism and the antioxidative effect of Gln.

Some studies have used Gln as a treatment for emotional and cognitive disorders caused by hypoxia. Hypoxia, which is a lack of oxygen, is known to affect synaptic signaling in the brain and increase inflammation, which can lead to mood disturbances and cognitive impairment [24]. In hypoxic conditions, the body’s natural synthesis of Gln may not be sufficient to meet the increased demand for immune system support, acid–base balance, ammonia transport, and neurotransmitter synthesis [24,79]. However, in both animals and humans, Gln supplementation attenuates inflammation and reduces the release of pro-inflammatory cytokines, such as TNF-α, IL-1β, and IL-6 [80,81]. These investigations focused on the antioxidative and anti-inflammatory effects of Gln rather than its neuropathological aspects.

Other clinical studies assessing the effects of Gln supplementation on emotional and cognitive function only reported behavioral improvements [23,25,26,27,28]. Early studies reported that Gln supplementation diminishes alcohol-induced cognitive function decrease [25,26] and increases IQ scores in children with mental deficiencies [27]. Additionally, Gln demonstrates antidepressant properties by showing therapeutic effects on asthenia in patients with depression, neurasthenia, and dissociative disorders [23].

## 5. Mechanism Studies of the Effects of Glutamine on Emotional and Cognitive Function

### 5.1. The Homeostasis of Glutamatergic Neurotransmission Is Essential for Normal Behaviors

To the best of our knowledge, the study by Lee et al. [31] was the first attempt to analyze the effects of Gln on neuropathological symptoms of depressive behaviors. In this study, researchers infused L-α-aminoadipic acid (L-AAA)—the astrocyte-specific toxin, which is a Glu analog that enters into astrocytes through Glu transporters, competitively inhibits GS, and induces astrocyte apoptosis [82,83]—into the prelimbic cortex (PLC) of mice and confirmed that astrocytic loss was sufficient to reduce Glu–Gln levels and induce depressive symptoms, such as anhedonia and despair behaviors [31]. Anhedonia and despair behaviors were analyzed using the sucrose preference test and forced swim test, respectively. The results show that depressive behaviors appeared on day 5 of L-AAA administration, when Glu–Gln levels decreased, and disappeared on day 10, when Glu–Gln levels were restored to normal. In addition, Gln infusion into the PLC of L-AAA-treated mice reversed the depressive behaviors to normal [31]. These data provide direct evidence that Glu–Gln deficiency in the PFC is a major cause of depressive behaviors.

Reduced Glu–Gln levels and depressive behaviors also appear when Gln synthesis or transport into neurons is disrupted with methionine sulfoximine (MSO, an irreversible inhibitor of GS) or α-methyl-amino-isobutyric acid (MeAIB, an irreversible inhibitor of Gln transporter SNAT2) [31]. In addition, GS inhibition by MSO infusion reduces the frequency of spontaneous excitatory postsynaptic currents (sEPSCs) from glutamatergic neurons in the mPFC [20]. These changes are normalized by Gln treatment on brain slices [20] or by direct infusion of Gln into the PLC [31], once again emphasizing the importance of Gln homeostasis in glutamatergic neurotransmission and depressive behaviors.

It was further shown that depressive behaviors are improved by specific optogenetic activation of glutamatergic neurons in the mPFC, indicating that the glutamatergic neurotransmission system in this region plays a key role in depression-related behavioral changes [20]. This is a step forward from previous studies that showed an improvement in depressive behaviors via non-specific activation of mPFC neurons [84,85,86,87].

Decreased glutamatergic activity has been commonly found in various animal models with emotional and cognitive disorders, including the L-AAA/MSO/MeAIB-treated mice described above [20,31]; chronic stress-induced depression and cognitive-impairment models [20,29]; a long-term hyperglycemia-induced social interaction disorder and depression model [88]; bis(2-ethylhexyl) phthalate (DEHP)-induced depression, learning and memory impairment, and social interaction deficit models [63,64]; a mouse model of spatial memory impairment induced by MSO treatment during synaptic development [22]; and a triple-transgenic (3xTg)-AD mouse model [32] (Figure 1). As hypoactive glutamatergic neurotransmission of various causes is commonly observed in different animal models, the maintenance of glutamatergic neurotransmission has emerged as an important strategy for developing new preventive and therapeutic agents against emotional and cognitive disorders. Therefore, further studies are required for understanding Gln homeostasis, given its key role in maintaining glutamatergic neurotransmission activity.

### 5.2. Glutamine Has Protective Effects on the Glu–Gln Cycle and Glutamatergic Neuronal Activity

**Figure 2:**
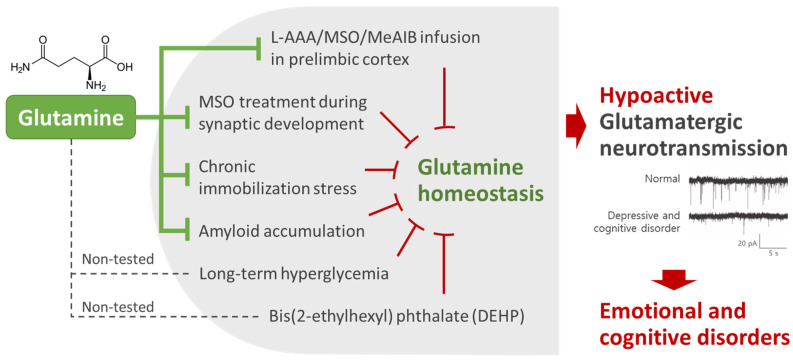
Glutamine (Gln) is essential for the homeostasis of glutamatergic neurotransmission. Changes in glutamate–Gln levels and decreased glutamatergic neurotransmission in the prefrontal cortex are commonly found in various animal models of emotional and cognitive disorders of different causes. These changes are reversed by Gln supplementation or direct infusion into the prelimbic cortex, suggesting that maintenance of Gln homeostasis and glutamatergic neurotransmission is important for emotional and cognitive function. L-AAA, L-α aminoadipic acid; MSO, methionine sulfoximine; MeAIB, α-methyl-amino-isobutyric acid.

Notably, unlike Gln, direct PLC infusion with Glu does not reverse depressive behaviors in L-AAA-induced astrocyte-deficit mice [31]. In addition, Glu treatment on brain slices does not reverse hypoactive glutamatergic neurotransmission [20]. In the latter study, both Glu and Gln treatments on brain slices in normal chamber solution significantly increased sEPSC frequency from glutamatergic neurons. However, when MeAIB inhibited Gln transport into neurons, neither Glu nor Gln treatment increased sEPSC frequency. Moreover, MSO-induced GS inhibition blocked the Glu-induced increase in sEPSC frequency. Taken together, these findings suggest that exogenous Glu must be converted to Gln by GS in astrocytes to be transferred to neurons and affect glutamatergic neurotransmission [20]. This is also consistent with previous studies suggesting that Glu must be in the form of its intermediate, Gln, in order to be transferred from astrocytes to neurons because the expression of Glu transporters in the presynaptic membrane is sparse [68,89]. Thus, Gln is believed to have greater potential as an antidepressant and cognition-improving candidate than Glu (Figure 2).

Accordingly, subsequent studies further explored the antidepressant and cognition-improvement effects of Gln and investigated the underlying mechanism for this phenomenon in animal models [20,29,30,90]. These studies mainly used chronic immobilization stress (CIS)-induced depression and cognitive impairment mouse models [29,91,92], which are more realistic than chemically induced depression models [31], to verify the antidepressant and cognition-improvement effects of Gln supplementation. To induce CIS, mice were subjected to a restrainer for 2 h per day for 15 consecutive days. Single-caged C57BL/6 male mice are mainly used for CIS models [92].

As observed in mice with MSO-induced depression, CIS-treated mice also exhibited low Glu–Gln levels, decreased GS activity without a change in expression level, and reduced sEPSC frequency in glutamatergic neurons within the mPFC accompanied by depressive behaviors. CIS-treated mice showed less preference for sucrose solution in sucrose preference tests and increased immobile time in tail suspension tests compared with non-stressed control mice [20,29,30,62,92]. Additionally, CIS model mice showed MCI in object recognition and object location recognition tests. CIS-treated mice showed less curiosity toward novel objects or objects that have been moved to a new location [29].

**Figure 3:**
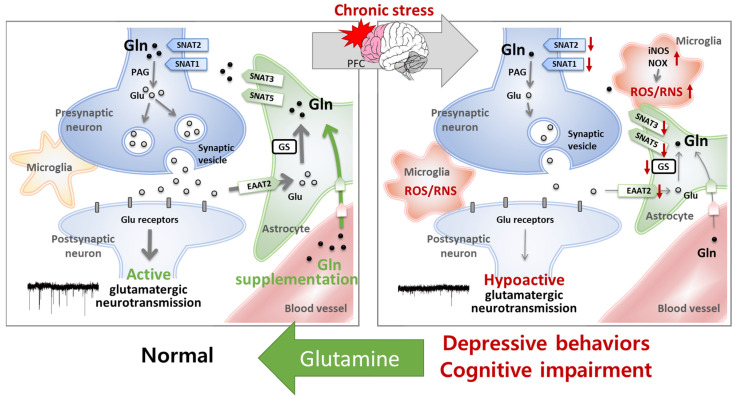
Glutamine (Gln) supplementation has antidepressive and cognition-improvement effects by protecting the homeostasis of glutamatergic neurotransmission. In a chronic immobilization stress (CIS)-induced depression and cognitive impairment mouse model, reduced glutamatergic neurotransmission was observed in the prefrontal cortex (PFC) along with low glutamate (Glu)—Gln levels, decreased Gln synthetase (GS) activity, and decreased expression levels of the Glu transporter excitatory amino acid transporter 2 (EAAT2) and Gln transporters in neurons (sodium-coupled neutral amino acid transporter [SNAT]1 and SNAT2) and in astrocytes (SNAT3 and SNAT5). Oxidative stress and pro-inflammatory changes were also observed in CIS mice. CIS also induces an increase in the levels of reactive oxygen/nitrogen species (ROS/RNS) and proteins related to ROS/RNS production in the PFC. These deleterious changes caused by chronic stress are reversed to normal levels by Gln supplementation, providing evidence that the restoration of Gln homeostasis through Gln supplementation has antidepressive and cognition-improvement effects. iNOS, inducible nitric oxide synthase; NOX, NADPH oxidase.

Further studies reported that expression levels of a Glu transporter (EAAT2) and several Gln transporters (SNAT1 and SNAT2 in neurons; SNAT3 and SNAT5 in astrocytes) were decreased in the mPFC in CIS-induced depressive mouse models, demonstrating a disruption of the Glu–Gln cycle by CIS [30]. Moreover, the width of the hippocampal pyramidal layer in the CA1 region, which is composed of glutamatergic neurons, was reduced by CIS [29], as reported in patients with MCI or AD [93,94]. However, these CIS-induced deleterious alterations were found to be ameliorated by Gln supplementation [20,29,30]. Mice provided with a Gln-supplemented diet containing 150 mg Gln/kg chow from 1 week before CIS until the end of the experiments did not show depressive behaviors or cognitive impairment after CIS. Reduced Glu–Gln levels, GS activity, Glu–Gln cycle protein expression levels, and sEPSC in the mPFC were also not observed in Gln-supplemented mice after CIS, nor were neuronal cell damages [20,29,30]. These results suggest that the disruption of Gln homeostasis is a main cause of CIS-induced hypoactive glutamatergic neurotransmission in the mPFC and eventually induces depressive behaviors and cognitive impairment (Figure 3).

### 5.3. Glutamine Has Antioxidant and Anti-Inflammatory Effects

The effect of Gln supplementation treatment on emotional and cognitive impairments has also been evaluated for its antioxidant and anti-inflammatory properties [29,32]. Notably, mice with CIS-induced depression and cognitive impairment showed increased blood corticosterone, a dominant stress hormone in rodents that triggers oxidative stress [20,29,30]. In addition, levels of reactive oxygen/nitrogen species (ROS/RNS) in plasma, the PFC, and the hippocampus increased in response to CIS [29], as did ROS/RNS levels in the plasma of 3xTg-AD mice at the MCI stage [32]. CIS also increased the expression of ROS/RNS production-related proteins, including inducible nitric oxide synthase (iNOS) and the NADPH oxidase subunits (p47phox and p67phox) and reduced synaptic puncta in the PFC and hippocampus [29]. The induction of iNOS and amyloid proteins was further observed in the PFC of MCI-stage 3xTg-AD mice, together with reduced sEPSC frequency [32]. In these studies, Gln supplementation was shown to restore cognitive function and reverse the deleterious changes responsible for oxidative stress and inflammatory responses [29,32] (Figure 3). Oxidative/nitrative stress is well known to induce inflammatory responses and affect neural plasticity [95,96]. Therefore, we suggest that the antioxidative and anti-inflammatory properties of Gln may contribute to improvements in depressive symptoms and cognitive impairments by influencing glutamatergic neuronal activity. However, there is still no direct electrophysiological evidence for a relationship between these Gln-associated antioxidative and anti-inflammatory properties and antidepressant and cognition-improvement effects. Thus, further investigation is necessary to clarify the underlying mechanisms.

## 6. Glutamine Supplementation Dosage

The appropriate dosage of Gln can depend on several factors, including the specific health condition, individual needs, and the form of Gln used (e.g., oral or intravenous). The dosages of Gln used in feeding trials vary depending on the specific research objectives and the population being studied. In clinical studies, Gln dosages often range from several grams to tens of grams per day. Although there are concerns about the effect of Gln on tumor growth, consuming 10–30 g of Gln per day in patients receiving chemoradiotherapy did not significantly affect tumor size or cancer-related clinical outcomes, proving the safety of Gln intake [97,98,99].

Gln supplementation in a dosage of 0.57 g/kg body weight per day (40 g/60 kg human per day) for 3 days improved the mood of marrow transplantation patients [28]. In another study, 1 g/day of Gln for 6 weeks increased the IQ scores of mentally deficient children [27]. The same dosage of Gln also reduced alcohol-induced cognitive decline [26].

However, Gln dosages used in depression and MCI animal models are much lower than those in the clinical studies. Kim and his colleagues provided mice with 450 μg/25 g mouse per day (18 μg Gln/g mouse body weight per day) for 3 weeks to 4 months [20,29,30,32], which is equivalent to approximately 90 mg/60 kg human per day (1.5 mg/kg human body weight) when accounting for the mouse–human differences in body surface area [100]. This amount of Gln was sufficient to show antidepressive and cognitive-improvement effects on mice, although the dosage was much lower compared with the clinical studies. This dosage of Gln reduced blood corticosterone levels, alleviated anhedonic and despair behaviors induced by chronic stress [20,30], and enhanced object cognitive functions in CIS-induced MCI models [29] and AD models [32]. Moreover, 450 μg Gln/25 g mouse per day activated glutamatergic neurotransmission in chronic stress [20] and AD mouse models [32]. Assuming that all the ingested Gln is absorbed into the blood and considering that the body’s blood volume is 7–8% of body weight, 90 mg/60 kg of Gln increases the blood Gln concentration by 128 μM. However, in related studies, Gln was not administered all at once but rather in divided dosages over a day. In addition, considering that absorbed Gln is rapidly metabolized in the body, the actual change in Gln concentration in the blood is likely much smaller than 128 μM. These results suggest that even slightly increasing blood Gln concentration can be greatly helpful in improving emotional and cognitive functions.

## 7. Conclusions

This review consolidates existing knowledge on the multifaceted roles of Gln in glutamatergic neurotransmission, emphasizing its potential as a therapeutic target for emotional and cognitive disorders. Gln homeostasis, especially in the PFC, plays an important role in the maintenance of the Glu–Gln cycle and glutamatergic neurotransmission associated with neuropathological conditions, including depressive and cognitive disorders. Recent studies have provided compelling evidence that the restoration of Gln homeostasis through Gln supplementation leads to antidepressive and cognition-improvement effects. This review highlighted the impact of Gln supplementation on synaptic signaling, oxidative stress, and inflammation, providing a foundation for its potential as a therapeutic agent. Additionally, the regulatory mechanisms of Gln concentration, distribution, and transport in the CNS were discussed, providing insight into the mechanisms of action and challenges of Gln supplementation. A further understanding of the mechanistic details underlying these Gln-associated antidepressant and cognition-improvement effects will provide opportunities to develop novel therapeutics for preventing and treating emotional and cognitive disorders.

## Data Availability

Not applicable.

## Abbreviations

^1^H-MRS	proton magnetic resonance spectroscopy
3xTg	triple-transgenic
AD	Alzheimer’s disease
AMPAR	α-amino-3-hydroxyl-5-methyl-4-isoxazole-propionate receptor
BBB	blood–brain barrier
CIS	chronic immobilization stress
CNS	central nervous system
CSF	cerebrospinal fluid
DEHP	bis(2-ethylhexyl) phthalate
EAAT	excitatory amino acid transporter
ECF	extracellular fluid
ECT	electroconvulsive therapy
GABA	γ-aminobutyric acid
Gln	glutamine
Glu	glutamate
Glx	Glu + Gln
GS	glutamine synthetase
iNOS	inducible nitric oxide synthase
KAR	kainate receptor
L-AAA	L-α-aminoadipic acid
MCI	mild cognitive impairment
MDD	major depressive disorder
MeAIB	α-methyl-amino-isobutyric acid
mGluR	metabotropic glutamate receptor
mPFC	medial prefrontal cortex
MSO	methionine sulfoximine
NMDAR	N-methyl-D-aspartate receptor
NMR	nuclear magnetic resonance
NOX	NADPH oxidase
PAG	phosphate-activated glutaminase
PFC	prefrontal cortex
PLC	prelimbic cortex
ROS/RNS	reactive oxygen/nitrogen species
sEPSC	spontaneous excitatory postsynaptic current
SNAT	sodium-coupled neutral amino acid transporter
VGLUT	vesicular glutamate transporter
